# FUTURE TIME PERSPECTIVE MODULATES AFFECTIVE REACTIVITY TO SOCIAL PROBLEMS DURING THE PANDEMIC

**DOI:** 10.1093/geroni/igac059.1960

**Published:** 2022-12-20

**Authors:** Elizabeth Zambrano Garza, Yoonseok Choi, Theresa Pauly, Denis Gerstorf, Christiane Hoppmann

**Affiliations:** University of British Columbia, Vancouver, British Columbia, Canada; University of British Columbia, Vancouver, British Columbia, Canada; University of Zurich, Zurich, Zurich, Switzerland; Humboldt Universität zu Berlin, Berlin, Berlin, Germany; University of British Columbia, Vancouver, British Columbia, Canada

## Abstract

Socioemotional Selectivity Theory posits that individuals with a limited future time perspective (FTP), prioritize emotionally meaningful, positive social interactions. Due to the high value placed on positive social interactions, individuals with a limited FTP might be particularly vulnerable to experiencing elevated negative affect when problems that involve other people do occur as compared to an experience of non-social problems. This project examined the role of FTP in modulating social problem–negative affect links during the pandemic, a time when people were particularly aware of their mortality, and thus their remaining time in life. This study used data from 150 Canadian adults (Mage =43 years, SD= 19, range 18-83, 78% women) who participated in two measurement bursts (one in 2020 and one in 2021). Participants provided FTP information at baseline and then repeatedly reported their negative affect and everyday problem characteristics in 10 consecutive daily diaries across both bursts. Results from multi-level models reveal a differentiated picture. The within-person association between social problem occurrence and elevated negative affect was significant among individuals with a more limited FTP. In contrast, at the between-person level, experiencing more social problems was associated with higher overall negative affect among those with a more extended FTP. To better understand these differential associations, follow-up analyses will explore if type of person involved in the problem and how the problem was handled matter for how they respond to social problems. Findings will be discussed in the context of the Strength and Vulnerability Integration model.

